# Evaluation of Prosthetic Marginal Fit and Implant Survival Rates for Conventional and Digital Workflows in Full-Arch Immediate Loading Rehabilitations: A Retrospective Clinical Study

**DOI:** 10.3390/jcm12103452

**Published:** 2023-05-13

**Authors:** Nicola De Angelis, Paolo Pesce, Marco De Lorenzi, Maria Menini

**Affiliations:** 1 Department of Surgical Sciences (DISC), Unit of Endo and Restorative Dentistry, University of Genova, Largo R. Benzi 10, 16132 Genova, Italy; 2 Dental Department, University Technology MARA, Sungai Buloh 40450, Malaysia; 3 Dental Department, University Trisakti, Jakarta 11440, Indonesia; 4 Department of Surgical Sciences (DISC), Unit of Prosthodontics and Implant Prosthodontics, University of Genova, Largo R. Benzi 10, 16132 Genova, Italy; paolo.pesce@unige.it (P.P.); maria.menini@unige.it (M.M.); 5 Private Practice, 15011 Acqui Terme, Italy; drmarcodelo@gmail.com

**Keywords:** dental implants, immediate loading, digital impression, full arch rehabilitation, temporary fixed bridge, guided surgery

## Abstract

Digital impression provides several advantages in implant prosthodontics; however, its use in full-arch rehabilitations, especially immediately after surgery, has yet to be validated. The aim of this study was to retrospectively analyse the fit of immediate full-arch prostheses, fabricated using conventional or digital impressions. Patients requiring a full-arch immediate loading rehabilitation were divided into three groups: T1 (digital impression taken immediately after surgery), T2 (Preoperative digital impression, guided surgery—prefabricated temporary bridge) and C (conventional impression taken immediately after surgery). Immediate temporary prostheses were delivered within 24 h after surgery. X-rays were obtained at the time of prosthesis delivery and at the 2-year follow-up. Primary outcomes were cumulative survival rate (CSR) and prosthesis fit. Secondary outcomes were marginal bone level (MBL) and patient satisfaction. One hundred and fifty patients were treated from 2018 to 2020, with 50 in each group. Seven implants failed during the observation period. The CSR was 99% for T1, 98% for T2 and 99.5% for C. A statistically significant difference in prosthesis fit was found among T1 and T2 vs. C. A statistically significant difference was found in the MBL between T1 and C. The outcomes of the present study suggest that digital impression is a viable alternative to conventional protocols for the realisation of full-arch immediate loading prostheses.

## 1. Introduction

Full-arch immediate loading rehabilitation is considered a predictable technique to rehabilitate completely edentulous patients or patients with a terminal dentition. Long-term studies validated this procedure [1,2,3,4], and a reduced number of implants was demonstrated to be sufficient as long as they are properly spread out for well spread for load distribution and sufficiently long to improve primary stability.

Immediate loading of dental implants is well accepted by patients since it reduces discomfort during the osseointegration period, avoids the need of a second surgical operation to uncover the implants, avoids the use of removable immediate temporary prostheses and reduces the number of doctor visits [5]. This approach allows patients to maintain function and aesthetic throughout the treatment, allowing them to maintain their usual daily activities and resulting in less social and work impact with important psychological benefits for the patient [6,7]. Conventional protocols for full-arch immediate loading rehabilitations require an analogic impression and occlusal registration immediately after implant placement. Rigid materials such as plaster demonstrated ideal results but were not scientifically evaluated for their trueness and accuracy and lead to optimal results for the fabrication of passive-fitting immediate frameworks [8].

More recently, digital impression gained popularity; however, its accuracy in full-arch immediate loading rehabilitations was not demonstrated [8,9,10]. While digital impression showed favourable outcomes, especially in the case of partial rehabilitations, clinical evidence is poor regarding trueness and accuracy in full-arch implant-supported rehabilitations [10] and it was mainly investigated in in vitro studies. In addition, impression in immediate loading presents peculiar challenges, since a completely edentulous arch is scanned when the flaps were just stitched and were still bleeding.

An alternative is the use of completely digital workflows that might fasten and simplify the clinical procedures while avoiding the need for taking an impression immediately after surgery. With a guided surgery approach, the immediate temporary prosthesis can be fabricated before surgery and delivered immediately after implant insertion, thereby decreasing patient discomfort.

Thus, the aim of the present study is to compare the fit and clinical outcomes of full-arch immediate loading prostheses fabricated using three different and currently used methods: conventional impression taken immediately after surgery, digital impression taken immediately after surgery and guided surgery and delivery of a prefabricated temporary bridge.

## 2. Materials and Methods

### 2.1. Study Design

The study was designed as a retrospective clinical study according to Strobe guidelines for cohort studies [11]. The participants were consecutively recruited from the patients visiting the private clinic of the principal investigator (N.D.A.) and divided into 3 groups (Test 1, Test 2 and Control) and followed up by the investigators, monitoring the outcomes for 2 consecutive years. All patients were asked to sign an informed consent with a full explanation of the procedure according to the Helsinki declaration for human rights. The procedures included in the present investigation did not include any experimental material or techniques. All the procedures were performed in accordance with the instruction of the regional ethical committee. All patients were informed that they were allowed to withdraw from the study at any time.

### 2.2. Inclusion and Exclusion Criteria

Any patient with a terminal natural dentition and requiring an implant-supported full-arch rehabilitation, being at least 18 years old with no restriction of race, gender and nationality was included in the study.

Exclusion criteria were:

- General contraindications to implant surgery;
- Immunosuppressed or immunocompromised patients;
- Irradiation in the head or neck area;
- Uncontrolled diabetes;
- Pregnant or lactating;
- Untreated periodontitis;
- Poor oral hygiene and motivation;
- Substance abuse;
- Heavy smokers (more than 10 cigarettes/day);
- Psychiatric disorders or unrealistic expectations;
- Acute infection in the site intended for implant placement;
- Unable to commit to 2-year follow-up post-loading;
- Under treatment or had previous treatment with intravenous amino-bisphosphonates;
- Participation in other clinical trials interfering with the present protocol;
- Sites, judged by the investigator, with a bone volume insufficient to guarantee at least 1.5 mm all around the implant.

### 2.3. Outcomes of the Procedure

Outcomes of the protocol were divided into main and secondary. Main outcome was related to the implant cumulative survival rate (CSR). Secondary outcomes were fit of the temporary prosthetic framework over the implant connections/abutments, evaluated immediately after prosthesis delivery; peri-implant marginal bone level evaluated over two years after the rehabilitation; and patient satisfaction.

### 2.4. Clinical Procedure

**Figure 1:**
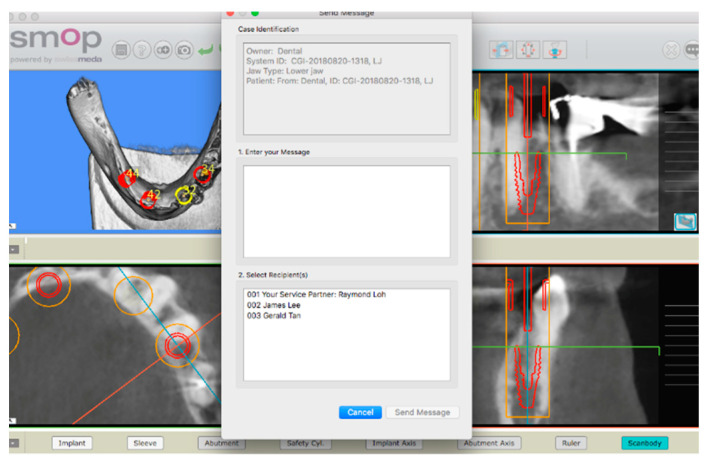
Planning of a case with SWISSMEDA AG OBERMÜHLE 8 6340 BAAR, SWITZERLAND software. Implant size and length can be changed as well as implant position and angulation. After completing the planning, the order of the surgical stent can be managed by the web site.

Before surgery, all the patients underwent professional oral hygiene and were carefully instructed on the oral maintenance before and after surgery. A digital impression, clinical photos and cone beam computed tomography (CBCT) were taken on the same day. The CBCT and standard triangulated language (STL) model of the arch selected for surgery were uploaded to software for surgical planning (SWISSMEDA AG OBERMÜHLE 8 6340 BAAR, SWITZERLAND provided by MS Company) and matched together to select the best implant size and length and plan the implant position (Figure 1).

All the surgical and prosthodontic procedures were executed by the same experienced clinician (N.D.A.) in a private dental clinic. Surgeries were performed under local anaesthesia (Articaine 40 mg/mL with Epinefrine 1:100,000). Extractions were carried out without flap elevation, and at least one tooth was left in order to match the preoperative impressions and the postsurgical ones and reproduce the occlusion.

**Figure 2:**
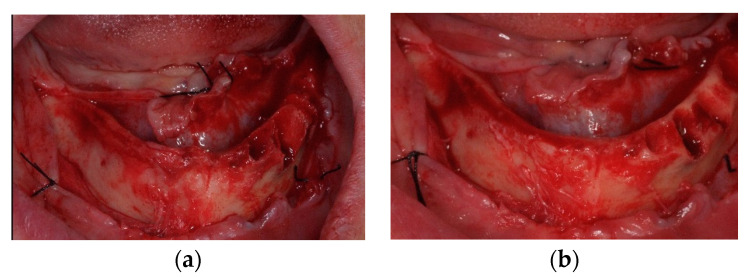
(**a**) Intraoral pictures of a mandibular case after teeth extraction and flap elevation; (**b**) a minimal ostectomy was performed in order to compensate for bone discrepancies.

A full thickness flap was raised from molar to molar, and flap elevation was extended on the buccal sides of the maxilla and the mandible, isolating and preserving the anatomical structures. Minimal ostectomy was performed when necessary to compensate for bone discrepancies (Figure 2).

**Figure 3:**
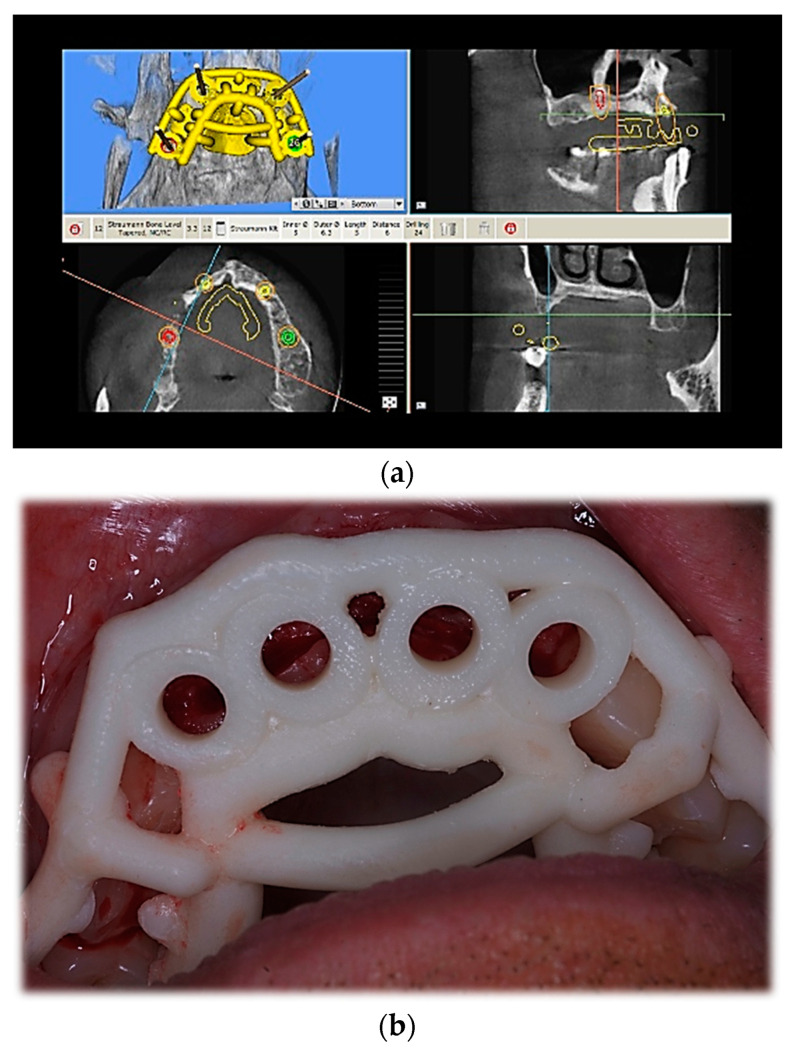
Intraoral picture of a patient of T2 group: (**a**) surgical stent design before printing; (**b**) placement of the surgical guide.

After flap elevation, the operator performed the site preparation with or without the printed surgical guide with the sequence of drills according to the implant system (Straumann^®^ Holding AG, Basel, Switzerland—BLT SLActive surface) as described in Figure 3. The operator was strictly forced to follow the provided protocol for the creation of the osteotomies.

**Figure 4:**
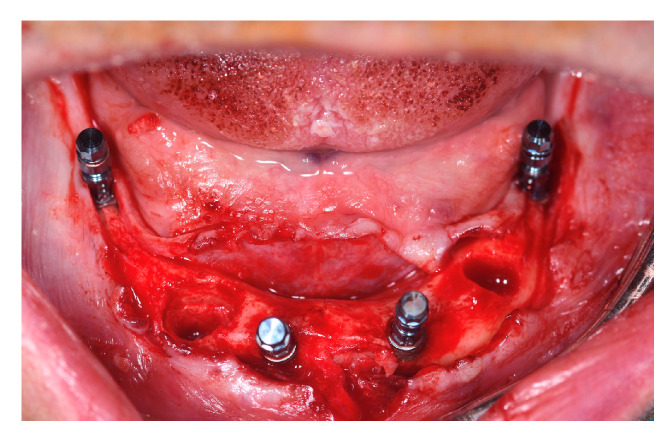
Intraoral picture of implant placement.

Implant sites for participants belonging to T1 and C were undersized, if needed, in order to reach the maximum primary stability by drilling bone, skipping the last dedicated drill in soft bone class 4. Implant placement followed the Columbus Bridge Protocol described in 2011 by Tealdo T. et al. [12]. The two distal implants were mesio-distally tilted and had a minimum length of 12 mm (Figure 4).

**Figure 5:**
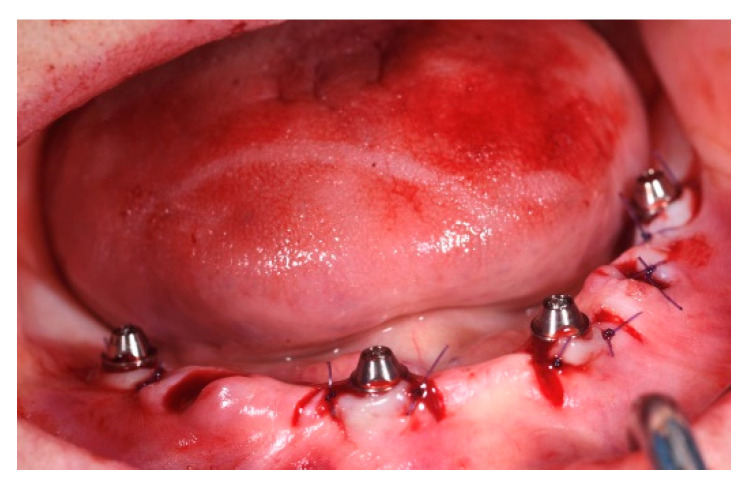
Abutment connection.

After implant placement, the multi-unit abutments were connected to the implants at 25 Ncm according to the following pattern:

- 0° or 17° on the anterior implants;
- 30° on the posterior tilted implant (Figure 5).

**Figure 6:**
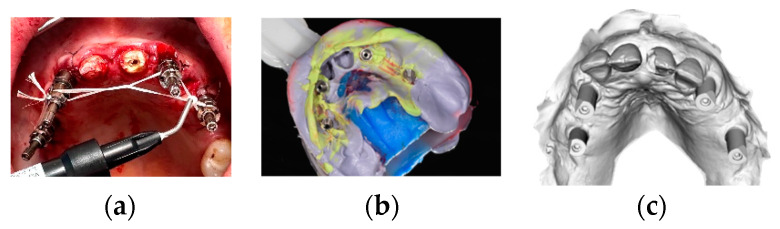
(**a**,**b**) Pick-up transfers tied together and PVS impression; (**c**) stl file of the digital impression.

Flaps were sutured with interrupted resorbable sutures (4/0 Vicryl^®^ Ethicon, Somerville, NJ, USA). In patients belonging to the T1 group, scan abutments were connected to the multi-unit abutments and a full-arch digital impression was taken using Trios 3 Shape (København K, Capital Region, 1060, Denmark). In patients belonging to the C group, pick-up transfers were connected on the platforms of the abutments and tied together with interdental floss as a frame for cyanoacrilate solidarisation (Periacryl High Viscosity^®^ GluStich, Delta, BC, Canada) or by using flowable light curing composite (Bulkfill^®^ Kyoto, Japan). Impressions were taken with standard plastic open trays and polyvinilsiloxane (PVS) material (Flexitime Regular and Heavy body^®^ Kulzer, D-63450 Hanau, Germany) (Figure 6).

**Figure 7:**
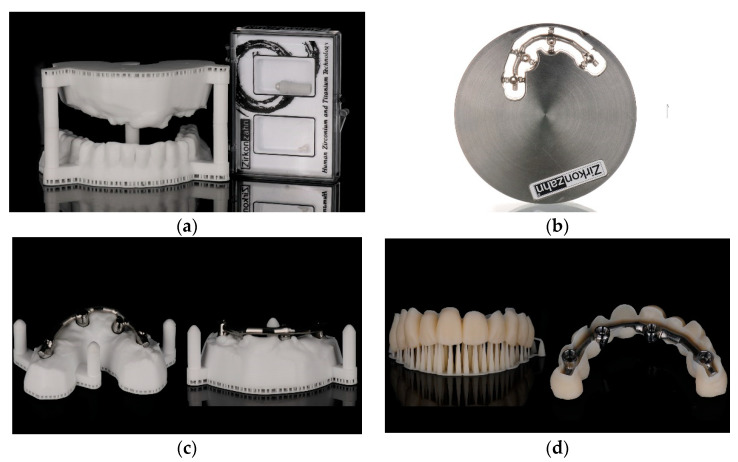
(**a**–**d**) A resin model was created for all patients in all groups, and the titanium milled bar was inserted and cemented into the acrylic teeth structure.

In groups T1 and C, after the impressions were taken, any remaining teeth were extracted, and patients were dismissed and scheduled for the delivery of the temporary bridge within 24 h after surgery from the surgical intervention for all groups, while patients belonging to the T2 group received the immediate temporary prosthesis immediately after surgery, that was obtained directly from the laboratory according to the surgical plan and the preoperative impression. In T2, the prosthesis was delivered together with the surgical guide before surgery. In all the groups, temporary bridges were made of printed acrylic resin and endowed with a titanium milled bar, with no cantilevered extensions (Figure 7).

After delivery of the fixed prosthesis, occlusion was carefully checked and corrected. All interferences were eliminated. A digital ortopanoramic X-ray was retrieved immediately after the delivery of the temporary bridge (time/month 1—t1). Patients were instructed not to brush for the first two weeks after surgery and to observe a soft diet for 30 days. Antibiotic therapy was administered to all participants through the use of Amoxicillin 875 mg+ Clavulanic Acid 125 mg twice/day for 6 days (in case of allergy Azithromicin 500 mg/day for 6 days) [13,14] and NSAID to control pain according to their needs. For the oral hygiene maintenance, Chlorexidine 0.20% was prescribed to be used at least during the first 30 days after surgery. Appointments were scheduled according to the following pattern: 10 days: suture removal, 15 days: check-up, 90 days: temporary bridges unscrewed and implant stability check, professional oral hygiene session (then repeated every 3 months), 120 days: final digital impression, 130 days: final prosthesis delivery and X-ray, 730 days (2 years—time/months 2-t2): check-up and X-ray.

### 2.5. Outcomes Evaluation

The implant cumulative survival rate (CSR) was evaluated in all the groups. Postoperative panoramic X-rays, obtained for all patients included in the investigation, were corrected and equalised by the software CorelDRAW Graphics Suite 2023^®^ (Ottawa, Canada) with a linear operation, expressed by the following equation:

$$feq\left(a\right)=Hi\left(a\right)\cdot \left(K-1\right):MN$$

where *M* and *N* are the selected dimensions of the image with pixels in the interval [0; *K* − 1] and then further magnified to a maximum of 300 dpi by Adobe Photoshop^®^ software (San Jose, CA, USA), and the bar connection on the platform of the abutment was resized and processed by ImageJ^®^ software (Rasband, W.S., ImageJ, U. S. National Institutes of Health, Bethesda, MD, USA), which allows the measures through the Analyse function.

**Figure 8:**
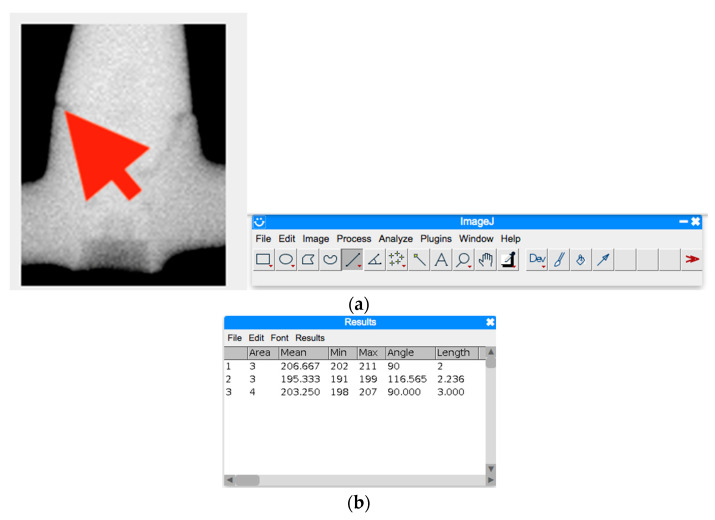
(**a**,**b**) X-rays were cropped in order to have only the connection bar/abutment, equalised and magnified. The red arrow indicates the gap between the bar and the abutment, which was measured. They were then imported on ImageJ which allowed us to measure the distance in pixels between one point and another. Measures were then converted to millimetres using the software (https://www.convertire-unita.info/convertire+Pixel+in+mm.php, accessed on 20 April 2023).

**Figure 9:**
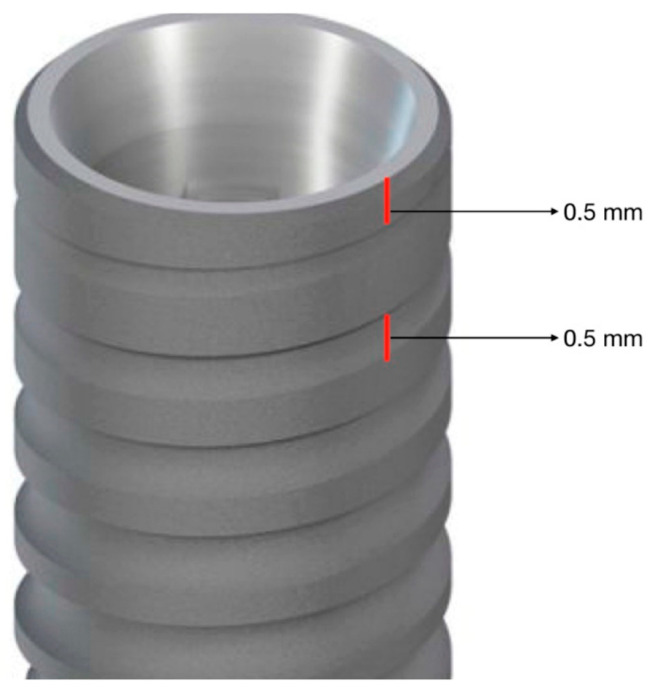
Schematic representation of the implants used in the study with indications of the distance between the platform and the first thread, as well as the distance between the threads.

As described in Figure 8, by the use of Image J, the distance between the prosthesis (upper line) and the abutment platform (lower line) was calculated using number of pixels and expressed in a linear function. Since there was no presence of irregular peaks, no further mathematical analysis was needed. Secondary outcomes were the marginal bone level and patient satisfaction. Marginal bone level was evaluated at time/months 1 (t1) and at time/months 2 (t2), calculating the distance between the implant platform and the most apical position of the bone on the mesial and distal side of each implant. As a reference for calculation, the distance between the implant platform and the first thread (0.5 mm), as well as the distance between the threads, was used (0.5 mm) (Figure 9).

At time/months 2 (t2), all the patients included in the study were requested to fill out a 4-question questionnaire, including the following questions:

1. How would you rate intraoperative discomfort?
2. How do you rate the discomfort during impression?
3. How do you rate the immediate delivery of the temporary prosthesis?
4. Overall judgment on the procedure

The responses were delivered using a visual analogue scale (VAS) and categorised as follows:

0 = nothing to report;
1–3 = minimal response;
4–6 = medium response;
6–10 = high response (very stressful).

### 2.6. Statistical Analysis

For the analysis, a sample composed of 150 observations was considered. Given a theoretical prevalence rate of 95%, the sample size analysis returned a minimum sample of 73 observations (considering a confidence interval of 95% and 5% margin of error). Three computer-generated restricted random lists were created. The implant was the statistical unit of the analyses. An intention-to-treat (ITT) analysis was used. R software (R Software Inc., San Francisco, CA, USA) was used for all tests. Differences in the proportion of patients with implant failures and complications (dichotomous outcomes) were compared between the groups, using the Fisher extract probability test. Differences of means at the implant level for continuous outcomes (gap bar/abutment and bone levels) between groups were compared by *t* tests. The Mann–Whitney U test was conducted to compare the medians of the three groups for patient satisfaction. Group distributions were tested to determine normality of the samples. Normality Jarque–Bera test was performed on all three samples, and the null hypothesis was not rejected. Since the samples were not normally distributed, the Mann–Whitney U test was applied since it is a nonparametric test that allows two groups or conditions to be compared without making the assumption that values are normally distributed. All statistical comparisons were conducted at the 0.05 level of significance.

## 3. Results

**Figure 10:**
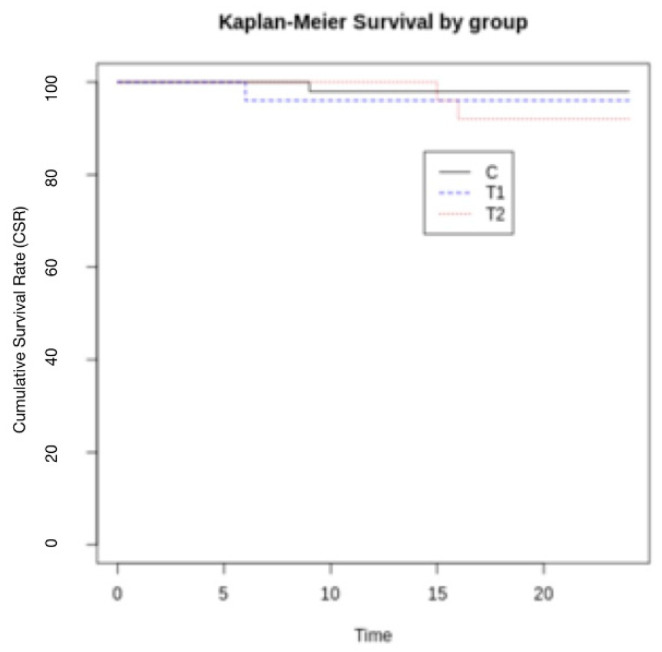
No statistically significant differences were present among the groups (*p* = 0.370).

One hundred and fifty patients, whose mean age was 55, were treated from 2018 to 2020, 50 for each group, 75 were males and 75 females. Seven implants failed during the observation period. Two implants failed in T1 before the final prosthetic delivery, 4 implants failed in T2 one year after final loading and 1 implant failed in C 6 months after the final prosthetic delivery. Implant failures were not connected to gender, neither or age. The final CSR was 99.0% for group T1, 98.0% for group T2 and 99.5% for group C. The Kaplan–Meier survival log is reported in Figure 10.

All the prostheses were considered clinically acceptable and immediately delivered without the need to take additional impressions. The mean gap between the bar and the abutment was 0.21 ± 0.10 mm, 0.28 ± 0.20 mm and 0.42 ± 0.20 mm for T1, T2 and C groups, respectively, with a statistically significant difference among T1 and C (*p* = 0.003) and between T2 and C (*p* = 0.002).

The marginal bone level mean differences are reported in Table 1. A statistically significant difference was present among T1 and C both at time/months 1 (*p* = 0.011) and at time/months 2 (*p* = 0.050). No statistically significant differences were found between T2 and control.

At the two-year follow-up visit (t2), the mean MBL was 0.35 ± 0.10 mm and 0.23 ± 0.80 mm in T1 for the maxilla and mandible, respectively; 0.37 ± 0.22 mm and 0.26 ± 0.14 mm in T2 in maxilla and mandible, respectively; and 0.4 ± 0.20 mm and 0.3 ± 0.12 mm in group C for maxilla and mandible, respectively. 

All groups of patients were equally satisfied by function (Mann–Whitney U test *p* = 0.880) and the aesthetics of their implant-supported rehabilitation (Mann–Whitney U test). All patients declared that they would undergo the same procedure again, and the most accepted part of the treatment (with the lowest mean and U value) was related to the immediate temporary dentition delivery, as displayed in Table 1.

## 4. Discussion

The results of the present study suggest that, even if all the herein described procedures led to a clinically acceptable fit of the prostheses, with optimal clinical outcomes at the 2-year follow-up, a statistically significant difference was found in the fit of the prostheses made following the three different techniques analysed.

Historically, a full-arch prosthesis supported by two distal tilted implants and two upright anterior implants were implanted free hand with the help of some devices useful in planning the tilting of the implant [15], and standard analogic impression techniques were employed. This allowed us to obtain an optimal implant and prosthesis cumulative survival rate and marginal bone loss in the medium term [2,4,15,16]. The results were confirmed over a 10-year period by Pera et al., who reported an implant cumulative survival rate of 93.25% and a mean bone loss of 2.11 mm [1].

Seven implant failures were observed throughout the duration of the study; however, two implants were lost in the T1 group before the final prosthetic delivery, which may be suggestive of a related non-osseointegration problem, while the other failures were observed after the final prosthetic delivery, which means at least 6 months after the implant placement. It is difficult to establish the exact causes of these complications, but a retrospective study published in the Journal of Clinical and Experimental Dentistry in 2020 evaluated the implant survival and success rates for All-on-4 procedures in a group of 104 patients. The authors found that the implant survival rate was 97.2%, while the success rate was 93.3%. In the present study, the final CSR was 99% for group T1, 98% for group T2 and 99.5% for group C, which is in agreement with the data reported by the other authors [17].

Moreover, a systematic review and meta-analysis published in the Journal of Clinical Periodontology in 2020 evaluated the implant survival and success rates of All-on-4 procedures in patients with severe bone loss. The authors found that the implant survival rate was 98.9%, while the success rate was 97.7% [18].

Similar outcomes were found in the present research. After a follow-up of 2 years in the T1 group, the CSR was 99% and the mean bone loss was 0.35 ± 0.1 mm and 0.23 ± 0.8 mm in the maxilla and mandible, respectively. This supports the statement that new digital technologies and procedures might help to simplify clinical workflows, maintaining the same accuracy of conventional protocols.

Impressions were taken with Trios 3 Shape intraoral scanner (Shape København K, Capital Region, 1060, Denmark) by using the continuous scanning technique, which saves time and reduces patient discomfort as it eliminates the need to pause and reposition the scanner multiple times. The accuracy of the Trios 3 Shape scanner is attributed to its high-resolution camera and advanced software algorithms, which can capture and process high-quality images quickly. The scanner also has a real-time feedback feature that allows the operator to adjust the scanning technique to ensure that the images captured are accurate. In terms of capturing accuracy, the Trios 3 Shape scanner was found to be highly accurate, with studies reporting accuracy levels of up to 99.7% [19].

Digital impression was proven to be a reliable method to impress single or partial implant rehabilitations; on the contrary, data on full-arch implant impressions are more contradictory [20,21]. Recent studies reported an increasing precision in the full-arch impression accuracy. Papaspyridakos et al., evaluating retrospectively digital vs. conventional full-arch implant impressions, reported that the 3D implant deviations found between the two groups (88 ± 24 μm) lie within the clinically acceptable threshold [22]. Similar results were reported in the present research, even if it must be highlighted that a higher discrepancy between the bar and the abutment was registered in the group treated with the guided surgery, when compared to digital impressions taken after surgery.

It should also be noted that the conventional impression in the present study was a polyvinylsiloxane impression with implant splinting with cyanolacrylate or composite material and showed the worst fit of all the prostheses compared to T1 and T2. The plaster impression used in other studies on full-arch immediate loading [1,3,8] was not evaluated in the present investigation.

Although compelling human evidence does not exist for clinical effects on peri-implant bone during the healing phase, a prosthesis misfit might induce noxious biomechanical stresses on the implants and prosthesis components and might favour screw loosening. For this reason, a very precise impression technique must be applied, aimed at achieving a clinically acceptable passive fit within 24 h after surgery. This is particularly important especially when using a screw-retained prosthesis, while a minimum misfit might be compensated in cemented prostheses due to the cement space. However, screw-retention is considered the best option by the authors for full-arch implant-supported restorations, since it facilitates the detection and management of possible complications, prosthesis relining and avoids the risk of cement-induced periimplantitis [5,6,7,8,9,10,11,12,13,14,15,16,17,18,19,20,21,22,23]. It is relevant to notice that the prosthetic framework is connected to the abutments, which are screwed onto the implants at the time of surgery; therefore, the possibility of complication and/or deformation of the internal connection of the implants, as described by Pandey C. in 2022, is not relevant when this technique is used [24].

Some limits of the present research must be acknowledged. Foremost, the fit was evaluated on panoramic x-ray, and this could have affected the outcome measures. The mean gap between the prosthesis and the abutment was quite high in the present study (0.21 ± 0.1 mm, 0.28 ± 0.2 mm and 0.42 ± 0.2 mm for T1, T2 and C groups, respectively). In a previous in vitro study on full-arch implant supported prostheses, the mean gap was lower than 30 µm, as measured by a stereomicroscope [10]. The different instruments applied for scanning and measurement, as well as the additional variables inherent with the clinical environment, might have affected the outcomes. Secondary, the number of included patients is quite high, but a bias might arise connected to the operator skills, since all surgeries and prosthodontics were performed by the same operator. It must be considered that a learning curve for both the clinician and the dental technician is essential to obtain the same outcomes.

## 5. Conclusions

The outcomes of the present investigation bring about promising steps to the clinical management of full-arch immediate rehabilitation protocols by merging a consolidated knowledge on the long-term survival of four dental implants, supporting a fixed full-arch prosthesis with the innovative digital impression technique and guided surgery approach, which shortens the operative times, increases the precision of the framework and is addressed to a future view of sustainable and eco-friendly systems.

## Figures and Tables

**Table 1:** List of questions for patient satisfaction questionnaire. * 0 = nothing to report; 1–3 = minimal response; 4–6 = medium response; 6–10 = high response to stress.

**Question**	Rate (Score 0 to 10) * Mean Value
How did you experience the intraoperative discomfort? (length of the procedure, physical discomfort)	3 (U = 1.5)
How do you evaluate the impression technique?	5 (U = 2.5)
How do you evaluate the immediate delivery of the temporary dentition?	0 (U = 0)
Overall judgment on the procedure	3 (U = 1.5)

## Data Availability

Not applicable.
